# The psychosocial impact of caring for children with Dravet Syndrome

**DOI:** 10.1016/j.ebr.2023.100619

**Published:** 2023-08-25

**Authors:** Rafael Salom, Luis Miguel Aras, Jessica Piñero, Jon Andoni Duñabeitia

**Affiliations:** a Centro de Investigación Nebrija en Cognición (CINC), Facultad de Lenguas y Educación, Universidad Nebrija, 28248 Madrid, Spain; b Asociación ApoyoDravet, 20009 San Sebastián, Spain; c Servicio Navarro de Salud-Osasunbidea, 31010 Navarra, Spain; d Fundación Salud Infantil, 03201 Elche, Spain; e AcqVA Aurora Center, Department of Languages and Culture, UiT The Arctic University of Norway, 9019 Tromsø, Norway

**Keywords:** Dravet Syndrome, Caregivers, Social impact, Psychosocial assessment

## Abstract

This study examined the psychosocial impact on parents of children affected by Dravet Syndrome (DS), a rare drug-resistant developmental encephalopathic epileptic syndrome which affects children at an early age and that involves severe cognitive, behavioral, and motor impairments. DS has a major negative impact on caregivers, mainly on their physical and mental health, and on their social relationships and economic resources. Similarly, it has been suggested that the quality of life of caregivers and children with DS is lower compared to the general population, especially because of the severe and frequent seizures suffered by the child, leaving caregivers with heavy burdens. The main aim of the current study was to assess in detail the psychosocial impact that having a child with DS represents for their parents or caregivers. To this end, a standardized assessment tool was used, and the results were compared to those of a control group. The results highlighted critical differences in most of the areas explored, revealing a marked difference between parents caring of children with DS and parents of normotypically developing children in the psychosocial wellness. This study provides important qualitative data to help us understand and identify the complexity of DS.

## Highlights

• Higher social burden scores are observed in caregivers of children with Dravet syndrome (DS) compared to the control group.
• Caring for a child with DS affects physical and emotional health of parents and their social relationships.
• Disruptive behaviors of children with DS create burdens and fatigue on caregivers.
• Caring for children with DS carries overwhelming financial burden.

## Introduction

The present study focuses on analyzing the quality of life of family members of individuals affected by Dravet Syndrome (DS) by means of exploring the impact of the condition in different psychosocial areas. DS, also known as polymorphic epilepsy or Severe Myoclonic Epilepsy of Infancy (SMEI), is a rare and severe genetic disorder characterized by developmental encephalopathy with pharmacoresistant persistent seizures with a prevalence of 1/20,000 to 1/40,000 [1], [2]. DS involves cognitive, behavioral, and motor deterioration and speech, mobility, learning, and sleep disorders [3], [5], and individuals affected by DS are at a high risk of sudden unexpected death in epilepsy (SUDEP), which generates great concern and anxiety in the immediate family context [6], [7].

The current DS treatment primarily focuses on controlling patients' seizures. However, these treatments are palliative and not curative in essence, and they can have significant side effects, including fatigue and cognitive or behavioral impairment. Additionally, administering these treatments can be challenging for caregivers due to their complexity [7], [8]. The challenges imposed by DS and its treatments have negative effects on primary caregivers, involving physical, mental, social relationships, and economic burdens [4], [6], [9], [10]. Primary caregivers of children with DS often experience high levels of emotional stress and interpersonal problems with other family members and their social environment [11], [12]. Furthermore, families often lack the necessary social support to cope with the challenges of the disease. The stigma associated with DS hinders the creation of an acceptable social environment due to the characteristic disruptive behavior issues associated with the syndrome [13]. Also, considering that patients with DS have unpredictable and frequent seizures, this generates a constant concern for the child’s physical well-being in caregivers, increasing anxiety and depression levels [7], [9].

Some previous studies have investigated how Dravet Syndrome affects caregivers' quality of life. These studies have mainly used a variety of quality-of-life measurement tools and online surveys. On the one hand, some studies have used specific assessment tools that exclusively focus on the affected children, such as the Pediatric Quality of Life Inventory [14]. On the other hand, some other studies have used more general tools, such as validated questionnaires aimed at the general population [4] or non-validated (online or telephone) surveys to obtain qualitative information on the quality of life of family members [9], [15], [16], [17], [18]. While these two approaches are necessary and informative, in the current study we aimed at filling the gap regarding the psychosocial impact on families with children with rare epileptic diseases by using a specific assessment tool designed for this purpose. In addition, for a more accurate and comprehensive assessment of the specific differential aspects of the psychosocial impact, and differently from most preceding studies, we included a control group for comparisons purposes. This allows us to determine whether the specific challenges and difficulties experienced by families of minors with Dravet Syndrome are unique to this population or common to families with or without the disease.

Given the limited information on the social impact of having a child with a low prevalence disease on families, this study aimed to explore the psychological and social consequences of DS on their families as primary caregivers. To this end, the Childhood Rare Epilepsy Social Impact Assessment (CRESIA) [19], a recently developed tool, was adapted and administered to a group of families with children and adolescents with DS, and the results were compared to those obtained from families of a normotypically developing sample of children.

## Methods and materials

### Participants

In the present study, 96 adults (76 women) participated, and the sample was divided into two groups. The first group consisted of a total of 48 adults (34 women), with an average age of 41.7 years (SD = 6.2). These individuals were parents of minors diagnosed with Dravet Syndrome (25 girls; average age of 8.8 years, SD = 4.8). The second group comprised a total of 48 adults (42 women), with an average age of 42 years (SD = 6.8). All the participants in this group were parents of minors without any diagnosed disease (25 girls; average age of 8.5 years, SD = 4.7). We found that the majority of caregivers who responded were female, which is consistent with the results of previous studies showing that there is an overrepresentation of the females in the areas of caregiving and early education. This bias is due to the cultural tendency to assign caregiving roles based on stereotypes closely linked to biological sex [20].

Additionally, information was collected on the professional and socioeconomic status of the respondents. Out of the 48 parents of minors with DS, 77% were active workers and 52% identified themselves as belonging to the lower-middle class. Similarly, out of the 48 parents of minors without any diagnosed disease, 81% were active workers and 58% identified themselves as belonging to the lower-middle class. The two groups were matched for the caregivers’ age (*t*(47) = 0.27, p = 0.79), the minors’ age (*t*(47) = 0.22, p = 0.82), the caregivers’ education level, (*t*(47) = 0.70, p = 0.48), and their social status (*t*(47) = 1.49, p = 0.14).

All parents provided written informed consent to participate in the study. This research was approved by the Ethics and Research Committees of Universidad Nebrija (protocol code UNNE-2022–006 approved on February 8, 2022).

### Procedure

All participants completed the Childhood Rare Epilepsy Social Impact Assessment (CRESIA) [19], which has a total of 371 items, ordered and presented according to the following domains: a) Social, b) Health, c) Psychological, d) Family, e) Stressors caused by the child, and f) Economic. All items were given and evaluated on a Likert-like scale from 1 (“not at all identified”) to 5 (“very identified”). The application time of the instrument is approximately 40 min, and it has good internal consistency, with a Cronbach's Alpha coefficient of 0.98. Regarding the evaluation of CRESIA in a healthy population, all items related to rare epileptic diseases and the economic scope for the control group were eliminated (46 of 371 items). Also, only families with normotypical children without any type of impairment that could interfere with the assessment were included in the control group, allowing for a more accurate comparison with the experimental group.

Once the corresponding informed consent was obtained, a detailed description of the study’s objectives and methodology was provided to each participant. Standardized instructions were provided for completing the CRESIA questionnaire.

The collected data were analyzed using the statistical software Jamovi [21]. Descriptive statistics and repeated measures ANOVA’s analysis were used to compare the data obtained from the two samples to identify significant differences between families with children with Dravet Syndrome and families of normotypically developing children (see Table 1).^1^

**Table 1:** Descriptive statistics of the results of the families with children with Dravet Syndrome (DS Group) and families with healthy normotypically developing children (NT Group).

Scale	Subscale	Group	Mean	Median	SD
Social		DS	3.09	3.20	0.51
		NT	2.41	2.23	0.54
	Perceived burden	DS	3.40	3.45	0.45
		NT	2.46	2.48	0.56
	Social support and self-concept	DS	2.76	2.88	0.77
		NT	2.34	2.19	0.65

Health		DS	2.84	3.03	0.95
		NT	2.35	2.13	0.77
	Self-perception of health	DS	2.54	2.80	0.93
		NT	2.17	2.00	0.84
	Emotional impact on physical state	DS	2.98	3.05	1.12
		NT	2.43	2.36	0.88

Psychology		DS	2.91	2.92	0.49
		NT	2.44	2.23	0.58
	Emotional state	DS	2.90	2.92	0.51
		NT	2.34	2.21	0.66
	Self-concept	DS	2.94	2.95	0.57
		NT	2.82	2.87	0.39

Family		DS	2.52	2.38	0.48
		NT	1.69	1.50	0.65
	Perceived family support	DS	2.51	2.33	0.53
		NT	1.74	1.40	0.82
	Family satisfaction	DS	2.12	2.00	0.91
		NT	1.67	1.50	0.63
	Impact on the family environment	DS	2.95	3.00	0.56
		NT	1.60	1.00	1.00

Stressors caused by the child		DS	2.72	2.76	0.61
		NT	1.79	1.74	0.50
	Social manifestations of the child	DS	3.45	3.60	1.17
		NT	1.88	1.80	0.73
	Behavioral manifestations of the child	DS	3.03	3.00	0.85
		NT	1.89	1.80	0.73
	Emotional manifestations of the child	DS	2.76	2.80	0.55
		NT	1.68	1.60	0.62
	Physiological and biological manifestations of the child	DS	2.24	2.25	0.85
		NT	1.68	1.50	0.70

## Results

**Fig. 1:**
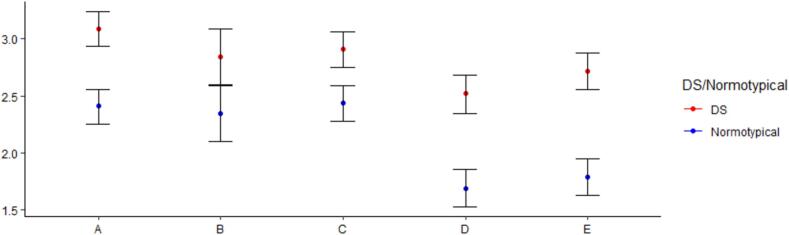
Psychosocial impact scores between families of minor with Dravet Syndrome and normotypical development. ***Note:*** A = Social; B = General Health; C = Psychological; D = Family; E = Stressors caused by the child.

The repeated measures ANOVA on the overall scores showed a significant main effect of Group (F(1,94) = 57.50, Mdiff = 0.68, p < 0.001, η2_generalized_ = 0.23), pointing to the existence of marked differences between caregivers of children with DS and of normotyopical children in their mean reported scores. Moreover, an expected significant main effect of the scale was found (F(4,376) = 29.98, p < 0.001, η2generalized = 0.139). Overall, in the post hoc test, significant differences were found in most of the comparisons between the scales. The mean scores corresponding to the average psychosocial impact in the Social, General Health, and Psychological scales did not differ from each other (all ts < 1.8 and p_bonferroni_ > 0.75). However, all these scales scored significantly higher than the Family and Stress caused by the child dimensions (ts > 3.75, p_bonferroni_ < 0.01), which did not differ from each other (t(94) = 2.41, p_bonferroni_ = 0.18). Finally, the interaction with the Scale factor was significant, showing that the differences between the groups were not homogeneous across domains (F(4,376) = 3.95, p = 0.004, η2_generalized_ = 0.021). Post hoc tests showed significant between-group differences in all the scales, with the effects being larger in the Social, Family, and Stress caused by the child scales (see Fig. 1 and Table 1): Social Mdiff = 0.68, t(94) = 6.32, p_bonferroni_ < 0.001; General health Mdiff = 0.5, t(94) = 2.79, p_bonferroni_ = 0.006; Psychological Mdiff = 0.5, t(94) = 4.26, p_bonferroni_ = 0.002; Family Mdiff = 0.82, t(94) = 7.01, p_bonferroni_ < 0.001; Stress caused by the child Mdiff = 0.93, t(94) = 8.18, p_bonferroni_ < 0.001.

Further ANOVAs were carried out to explore potential differences in each of the subscales of each scale. The interaction between the Subscale and the Group factor in the analysis of the results of the Social scale was significant, (F(1,94) = 16.00, p <.001, η2_generalized_ = 0.044), showing that while the main differences between the two groups existed in all subscales, they were larger in some of the subscales (Perceived burden t(94) = 9.00, p <.001; Social support and self-concept t(94) = 2.87, p =.005; see Table 1). A parallel analysis of the data from the Health scale showed no significant interaction between the factors (F(1,94) = 0.95, p <.33, η2_generalized_ = 0.002), thus suggesting that the differential effects between the groups held constant across subscales (Emotional impact on physical state t(94) = 2.69, p = 0.008; Self-perception of health t(94) = 2.03, p = 0.046). The ANOVA on the data from the Psychological scale showed a significant interaction between the two factors (F(1,94) = 10.70, p =.001, η2_generalized_ = 0.04), showing differences between the groups in the Emotional state (t(94) = 4.59, p < 0.001), but not in the Self-concept subscale (t(94) = 1.15, p = 0.25). The analysis of the Family scale also showed a significant interaction between the factors (F(2,188) = 15.50, p < 0.001, η2_generalized_ = 0.042), suggesting greater between-group differences in some of the subscales than others (Impact on family environment: t(94) = 8.09, p < 0.001; Perceived family support: t(94) = 5.47, p < 0.001; Family satisfaction: t(94) = 2.82, p = 0.006). Finally, the ANOVA on the scale corresponding to the Stressors caused by the child interaction was also found significant (F(3,282) = 8.14, p < 0.001, η2_generalized_ = 0.05), and post hoc analyses demonstrated that the magnitude of the between-group difference was larger in some subscales than in others, with all the pairwise contrasts still yielding significant differences (Emotional manifestations of the child: t(94) = 8.89, p < 0.001; Social manifestations of the child: t(94) = 7.87, p < 0.001; Behavioural manifestations of the child: t(94) = 7.01, p < 0.001; Physiological and biological manifestations of the child: t(94) = 3.56, p < 0.001).

**Fig. 2:**
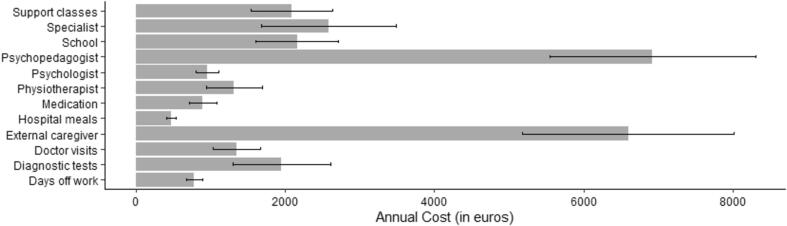
Annual economic costs for families of children with Dravet Syndrome.

Finally, the average economic cost of caring for a child with Dravet syndrome was studied. For this purpose, quantitative data from the Economic scale were analyzed for the caregiver group, showing an average annual direct cost of €25,819 for expenses incurred because of the child's illness (e.g., doctors, drugs, external caregivers). On the other hand, an average annual indirect cost of €2,280 is observed, referring to expenses that are not related to the child's illness but to the consequences of the illness on the family (e.g., psychologists and physiotherapists for any family member). The annual costs were further analyzed considering each of the budget items and is presented in Fig. 2.

## Discussion

The present study aimed to explore the psychosocial impact of Dravet Syndrome on families as primary caregivers. Different quantitative measures were used to assess caregivers’ quality of life-related to health, psychological status, family life, and child-related stress, and the data gathered were compared to those obtained from a sample of parents of healthy children. The results indicated that families of children with Dravet Syndrome experience a significant psychosocial impact as compared to families of children who develop normotypically.

Firstly, families of children with Dravet Syndrome were found to experience the highest levels of impact in the social domain, both perceived social burden and problems with social support and self-concept. These findings suggest that caring for a child with Dravet Syndrome leads to interpersonal relationship problems and social stigma. The second area with the highest level of impact was the general health of families. Their health was mainly affected by the high physical burdens and fatigue associated with caring for a child with a rare epileptic disorder. Thirdly, these results highlighted a significant impact in the psychological domain, directly affecting caregivers’ emotional state. Primary caregivers experience high levels of stress, anxiety, worry, and hopelessness, as well as low levels of meaning in life, self-concept, and personal growth. Fourthly, a high impact caused by the child's emotional, behavioral, and biological reactions was evidenced. Altogether, the child’s disruptive behaviors, basic needs, sleeping or feeding difficulties, and sporadic seizures all yield high levels of prolonged stress for the primary caregivers. Finally, family life or domain was also found to be significantly affected. Parents and caregivers of children with DS reported feeling lonely and receiving little help and support from their immediate family environment, which in turn exacerbates the psychosocial distance to parents of healthy children.

These findings are consistent with previous studies that have demonstrated the complexity of caring for a child with Dravet Syndrome. DS can make it difficult to form and maintain healthy social relationships [6], [13] and can increase emotional dysregulation [12], yielding problems with close family members [11], [13], as well as causing a high physical burden for caregivers [6], [9]. In addition, the physical, psychological, and behavioral conditions related to DS can generate a great deal of stress in the family [7], [9], [10], and the current results offer valuable insight into this by detailing the specific areas and subareas in which the psychosocial impact of caring for a child with DS is more clearly seen.

These results show that parents and primary caregivers of children with DS obtained significantly higher scores on all the scales assessed as compared to caregivers of healthy children, suggesting that the cost in terms of decreased quality of life and increased negative psychosocial impact extends to all the studied domains. The least pronounced difference was observed on the scale related to the general physical health of the caregivers, pointing to their mental and cognitive health as the key target of the intervention actions, as a less visible manifestation of the spillover effects of having a child with a rare disease such as DS.

Finally, the large economic burden of caring for a child with DS is worth noting. The direct and indirect expenses related to the child's disease were on average around 28,000 euros per year. This amount is surprisingly close to the average annual salary in Spain, set around 25,000 euros [22], and these results provide evidence that the cost associated with caring for a child with a rare disease represents a sometimes unsurmountable economic load for the family. These data align with preceding studies that emphasize the exaggerated costs related to DS [10] and suggest that the economic burden may represent one of the most relevant underlying reasons for the impoverished quality of life of families of children with Dravet Syndrome [12].

In conclusion, early assessment tools represent a promising avenue for developing evidence-based intervention protocols targeting families and primary caregivers of children with Dravet Syndrome. The psychosocial impact of Dravet Syndrome extends to the closest relatives with pervasive effects on different domains. These results highlight the need for new psychosocial treatment, prevention tools and policies to financially assist caregivers.

## Ethical Statement

The study was conducted in accordance with the Declaration of Helsinki and approved by the Ethics Committee of Universidad Nebrija (protocol code UNNE-2022-006 and approved on 8 February 2022) for studies involving humans.

## CRediT authorship contribution statement

**Rafael Salom:** Methodology, Formal analysis, Investigation, Data curation, Writing – original draft. **Luis Miguel Aras:** Conceptualization, Software, Resources, Writing – review & editing, Project administration. **Jessica Piñero:** Software, Investigation, Resources, Writing – review & editing. **Jon Andoni Duñabeitia:** Conceptualization, Methodology, Software, Resources, Writing – review & editing, Supervision, Project administration, Funding acquisition.

## Declaration of Competing Interest

The authors declare that they have no known competing financial interests or personal relationships that could have appeared to influence the work reported in this paper.

## Footnotes

1 The subscales corresponding to the physical limitations at work, work stress and work self-concept were excluded from the analysis given that the sample was not entirely composed of active working individuals.
